# Clinical and immunological outcomes of SARS-CoV-2 infection in patients with inborn errors of immunity receiving different brands and doses of COVID-19 vaccines

**DOI:** 10.5578/tt.20239705

**Published:** 2023-09-22

**Authors:** E. KARABİBER, Ö. ATİK, F.M. TEPETAM, B. ERGAN, A. İLKİ, E. KARAKOÇ AYDINER, A. ÖZEN, F. ÖZYER, S. BARIŞ

**Affiliations:** 1 Division of Adult Immunology and Allergy, Department of Chest Diseases, Marmara University Pendik Training and Research Hospital, İstanbul, Türkiye; 2 Division of Adult Immunology and Allergy, Department of Chest Diseases, Süreyyapaşa Training and Research Hospital, İstanbul, Türkiye; 3 Department of Medical Microbiology, Marmara University Faculty of Medicine, İstanbul, Türkiye; 4 Department of Pediatric Allergy and Immunology, Marmara University Faculty of Medicine, İstanbul, Türkiye; 5 İstanbul Jeffrey Modell Diagnostic and Research Center for Primary Immunodeficiencies, İstanbul, Türkiye; 6 Işıl Berat Barlan Center for Translational Medicine, İstanbul, Türkiye

**Keywords:** inborn errors of immunity, the Pfizer/BioNTech BNT162b2, Sinovac, SARS-CoV-2, COVID-19 vaccines, immün sistemin doğuştan gelen kusurları, Pfizer/BioNTech BNT162b2, Sinovac, SARS-CoV-2, COVID-19 aşıları

## Abstract

**ABSTRACT:**

Clinical and immunological outcomes of SARS-CoV-2 infection in patients with inborn errors of immunity receiving different brands and doses of COVID-19 vaccines

**Introduction:**

Vaccines against severe acute respiratory syndrome-coronavirus-2 (SARS-CoV-2) provide successful control of the coronavirus-2019 (COVID-19) pandemic. The safety and immunogenicity studies are encouraging in patients with inborn errors of immunity (IEI); however, data about mortality outcomes and severe disease after vaccination still need to be fully addressed. Therefore, we aimed to determine the clinical and immunological outcomes of SARS-CoV-2 infection in patients with IEI who have received vaccination.

**Materials and Methods:**

Eighty-eight patients with a broad range of molecular etiologies were studied; 45 experienced SARS-CoV-2 infection. Infection outcomes were analyzed in terms of genetic etiology, background clinical characteristics, and immunization history, including the type and number of doses received and the time elapsed since vaccination. In addition, anti-SARS-CoV-2 antibodies were quantified using electrochemiluminescent immunoassay.

**Results:**

Patients were immunized using one of the three regimens: inactivated (Sinovac, Coronavac®), mRNA (BNT162b2, Comirnaty®, Pfizer-Biontech), and a combination. All three regimens induced comparable anti-SARS-CoV-2 IgG levels, with no differences in the adverse events. Among 45 patients with COVID-19, 26 received a full course of vaccination, while 19 were vaccine-naive or received incomplete dosing. No patients died due to COVID-19 infection. The fully immunized group had a lower hospitalization rate (23% vs. 31.5%) and a shorter symptomatic phase than the others. Among the fully vaccinated patients, serum IgM and E levels were significantly lower in hospitalized patients than non-hospitalized patients.

**Conclusion:**

COVID-19 vaccines were well-tolerated by the IEI patients, and a full course of immunization was associated with lower hospitalization rates and a shorter duration of COVID-19 symptoms.

## Abstract (tr)

**ÖZ:**

İmmün sistemin doğuştan gelen kusurları olan hastalarda farklı marka ve dozlarda uygulanan SARS-CoV-2 aşısı sonrası SARS-CoV-2 enfeksiyonunun klinik ve immünolojik sonuçları

**Giriş:**

Şiddetli akut solunum sendromu koronavirüs-2 (SARS-CoV-2) aşıları koronavirüs (COVID-19) pandemisinin başarılı şekilde kontrol edilmesinde etkili olmuştur. İmmün sistemin doğuştan gelen kusurları (IEI) olan hastalarda aşıların güvenilirlik ve immünojenite çalışmaları ümit verici sonuçlar vermis olsa da aşı sonrası mortalite sonuçları ve hastalık şiddeti hakkındaki veriler henüz kısıtlıdır. Bu nedenle SARS-CoV-2 aşıları uygulanan IEI hastalarında SARS-CoV-2 enfeksiyonunun klinik ve immünolojik sonuçlarını değerlendirmeyi amaçladık.

**Materyal ve Metod:**

Kırk beşi SARS-CoV-2 enfeksiyonunu geçiren, farklı moleküler etiyolojiye sahip 88 IEI hastası çalışmaya dahil edildi. Enfeksiyon sonuçları, altta yatan genetik etiyoloji, klinik özellikler, uygulanan aşı doz sayısı, aşı markası ve aşılamadan hastalık oluşana kadar geçen süreye göre analiz edildi. Ayrıca anti-SARS-CoV-2 antikor düzeyleri elektrokemilüminesan yöntemi ile ölçüldü.

**Bulgular:**

COVID-19 geçiren 45 hastadan 26’sı tam doz aşılı iken, 19’u eksik doz ya da hiç aşı olmayan hastalardan oluşmaktaydı. Hastaların aşı şeması, inaktif (Sinovac, Coronavac®), mRNA (BNT162b2, Comirnaty®, Pfizer-BioNTech) ve iki aşının kombinasyonu olmak üzere üç farklı grupta değerlendirildi. Her üç grupta da benzer anti-SARS-CoV-2 antikor düzeyleri saptandı ve yan etki profili benzerdi. Tüm gruplarda COVID-19 enfeksiyonu ile ilişkili ölüm olmadı. Tam doz aşılı grupta hastane yatış oranı (%23’e karşı %31,5) ve semptomatik gün sayısı diğer gruba göre daha düşük idi. Tam doz aşılı olup hastane yatışı olanlarda serum IgM ve IgE düzeyleri hastaneye yatmayanlara kıyasla anlamlı olarak düşük saptandı.

**Sonuç:**

IEI hastalarında tam doz SARS-CoV-2 aşılaması iyi tolere edilir ve daha düşük oranda hastane yatışı ve daha az COVID-19 semptom süresiyle ilişkilidir.

## INTRODUCTION

During the span of three years since the onset of the coronavirus disease-2019 (COVID-19) pandemic, a cumulative total of 689.322.592 individuals have been confirmed to be infected with the SARS-CoV-2 virus, leading to more than 6.883.222 reported deaths attributed to the infection ( 1 ). Throughout the pandemic, numerous variants emerged, including the D614G mutant, UK/alpha (B.1.1.7), South Africa/beta (B.1.351), Brazil/gamma (B.1.1.248), India/delta (B.1.617), and most recently, multiple countries/ omicron (B.1.1.529) ( 2 ). Recently some different omicron variants have been spreading ( 3 , 4 ). While most individuals managed to recover from infections, a distinct subgroup of patients marked by advanced age and underlying conditions such as obesity, hypertension, coronary artery disease, malignancy, immunodeficiency, and renal and pulmonary disorders, are considered to be at a heightened risk for severe and unfavorable outcomes of COVID-19 ( 5 , 6 , 7 , 8 ).

The impact of SARS-CoV-2 on immunologic response is yet to be fully understood ( 9 , 10 , 11 ). Unlike other viruses, SARS-CoV-2 can escape from the innate immune system during the asymptomatic phase ( 11 ). It is known that anti-interferon antibodies contribute to disease severity ( 12 ). Complement system responses exacerbate COVID-19, and natural killer (NK) cell responses are compromised during the infection ( 13 ). The adaptive immune system is also disturbed, and persistent lymphopenia is a poor prognostic marker of severe disease. Despite the production of antibodies against SARS-CoV-2, it has been observed that in many deceased COVID-19 patients, the presence of antibodies was inadequate to provide protection against severe illness and failed to effectively neutralize the virus ( 14 ). Furthermore, effective early-T cell responses are associated with milder disease, and exaggerated or inadequate T-cell responses may lead to poor outcomes ( 15 , 16 , 17 , 18 , 19 ).

Patients with inborn errors of immunity (IEI) may exhibit heightened susceptibility to more severe COVID-19 infections. However, the specific subtypes of IEI play a crucial role in shaping the course of the disease ( 8 , 20 ), which makes it challenging to provide overarching recommendations for this particular population. It is well-known that patients with common variable immunodeficiency (COVID-19) can experience severe infection requiring intensive care; however, there are inconsistent results among patients ( 21 , 22, , 23 , 24 ).

Vaccination against COVID-19 is the mainstay of protecting from severe disease and terminating the COVID-19 pandemic. The effectiveness of vaccines in preventing disease following exposure to SARS-CoV-2 ranges from 50% to over 90%. However, the efficacy in preventing severe disease has been demonstrated to be nearly 95% ( 25 , 26 ). In our country, two vaccines are currently available for use: a recently introduced mRNA vaccine (BNT162b2, Comirnaty®, Pfizer-BioNTech) and an inactivated vaccine (Sinovac, CoronaVac®, Vero-cell) ( 27 ). Vaccination against SARS-CoV-2 has been endorsed by healthcare experts and has been demonstrated to be both safe and effective for individuals with IEI, who are also being prioritized globally ( 28 , 29 ). However, most data about the efficacy and safety of the vaccines are gathered from trials performed on healthy individuals. Limited data are available concerning the immunogenicity of SARS-CoV-2 vaccines and the subsequent outcomes of COVID-19 infection among vaccinated individuals with IEI. Due to the underlying immunological deficiencies, patients with IEI typically exhibit reduced or even absent vaccine responses ( 30 ). While some recent studies have indicated favorable tolerance and immunogenicity, further research is necessary to provide a comprehensive understanding ( 31 , 32 , 33 ).

Studies investigating the immunogenicity and efficacy of SARS-CoV-2 vaccines in patients with IEI have shown diminished levels of SARS-CoV-2 specific IgG and T-cell responses in comparison to the general healthy population (30-75% and 50-70% versus 95-100%). Additionally, patients exhibited lower titers of SARS-CoV-2 specific IgG, reduced efficacy in virus neutralization, and decreased magnitude of T-cell responses when compared to healthy donors. Notably, patients with low serum IgG, IgA levels, and those of older age demonstrated poorer vaccine responses ( 34 ).

In this study, our objective was to assess the clinical outcomes of SARS-CoV-2 infection in patients with IEI who have completed the full course of vaccinations, and to compare these outcomes with those of vaccine-naive and/or incompletely vaccinated patients. In addition, our study also assessed the efficacy and seroconversion rate of different vaccine regimens in patients with IEI.

## MATERIALS and METHODS

Patients with IEI were recruited from two different centers in İstanbul, Marmara University, Pendik Training and Research Hospital, and Süreyyapaşa Training and Research Hospital. IEI patients were eligible for the study entry if they were

1) Over 18 years old and

2) Vaccinated for SARS-CoV-2 either with inactivated or mRNA and/or encountered SARS-CoV-2 infection even if vaccinated or unvaccinated. IEI was diagnosed according to the European Society of Immunodeficiency clinical working party criteria and the International Union of Immunological Societies (IUIS) classification ( 35 , 36 , 37 ). Vaccination status was defined as full course vaccination (two or more doses) and incomplete vaccination (vaccine-naive or one dose of immunization). Probable COVID-19 variants were determined according to COVID-19 infection time which was in widespread global circulation at the time stated by World Health Organization (WHO). The severity of SARS-CoV-2 infection was defined using criteria according to WHO interim COVID-19 guidelines.

We collected a comprehensive dataset encompassing demographics, detailed clinical features with baseline therapies [prophylactic antibiotic usage, IgG replacement therapy (IgRT), immunosuppressive drugs], and immunological parameters (serum-IgG concentrations, lymphocytes enumeration and subsets, vaccination status, and infection time of SARS-CoV-2 and other laboratory assessment). SARS-CoV-2 infection was diagnosed by positive reverse transcription polymerase chain reaction (RT-PCR).

Data on COVID-19 infection was collected using a structured questionnaire, including contact history and COVID-19-related symptoms. Patients were also reviewed for the duration of hospitalization, treatment regimens during hospitalization or at home, and outcomes of COVID-19 infection.

Blood samples for anti-SARS-CoV-2 antibodies were collected before the subsequent dose of intravenous IgRT and at any time in patients who received subcutaneous IgRT (SCIG). An anti-SARS-CoV-2 S assay kit (Elecsys® Anti-SARS-CoV-2 S kit, Roche Diagnostic, USA) was used to detect antibodies. This is an electrochemiluminescent immunoassay for detecting antibodies to SARS-CoV-2 nucleocapsid (N) protein and performed on the Cobas® e401 analyzer. The antigens within the reagent capture predominantly anti-SARS-CoV-2 specific-IgG but also anti-SARS-CoV-2 specific-IgA and IgM. Serum samples were tested in accordance with the manufacturer’s instructions and results greater than 0.8 U/mL were categorized as seropositive. We assessed the antibody responses at 1/10 of diluted serum samples.

The study protocol was approved by the local ethics committee of our hospital with decision number 210. All participants provided written informed consent.

### Statistics

Median and interquartile range (IQR) values for continuous variables and the frequency and percentage for the categorical variables were calculated. Differences between ordinal data were evaluated with the Mann-Whitney U test and the Kruskal-Wallis test. Categorical variables were evaluated with the two tailed Chi-square or Fisher’s exact tests. Correlation tests were assessed with the Spearman’s correlation test. Statistical analyses were done using IBM SPSS 25 (SPSS Inc, Chicago, III) and GraphPad Prism 8 (GraphPad Software Inc. San Diego, California, USA). Differences in values were considered significant at a p-value of <0.05.

## RESULTS

**Figure 1:**
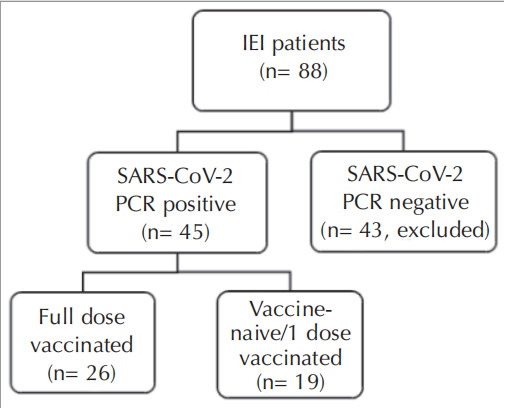
The study design and enrolled IEI patients.

A total of 88 IEI patients were enrolled in this study. Among our IEI cohort, 45 patients (51.1%) had encountered SARS-CoV-2 infection, and no deaths occurred. The study design is shown in Figure 1 . The median age of participants was 35 years (IQR= 25.5- 40), and 53.3% were male. Patients’ demographic characteristics, vaccination status, diagnosis of IEI, hospitalization, and other features are summarized in Table 1 .

**Figure 2:**
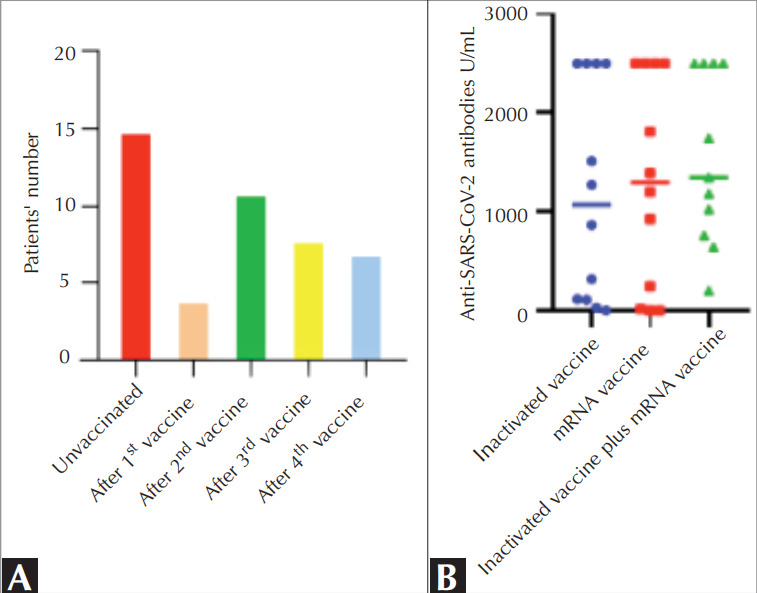
Vaccination rates and responses among IEI patients (A,B). Vaccination status at COVID-19 infection time (A). Titers of anti-SARS-CoV-2 antibodies in COVID-19-positive patients with IEI (B).

The median interval between the day of the last vaccination and SARS-CoV-2 infection was 85 days (IQR= 28.5-161.25), and the majority of patients received at least two doses of vaccination during exposure to SARS-CoV-2 ( Figure 2 A ). The median time interval between the day of the final vaccine dose and the sampling of anti-SARS-CoV-2 antibodies was 133 days (IQR= 79-229). The median time interval between the day of SARS-CoV-2 infection and the testing for anti-SARS-CoV-2 antibodies was 140.5 days (interquartile range= 62.7-347.7).

**Figure 3:**
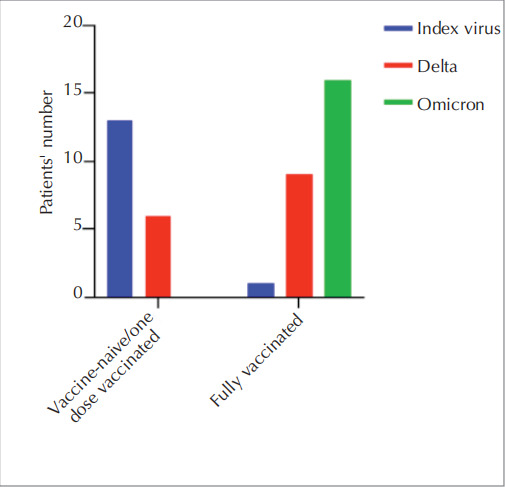
The initial total number of patients, along with the count of patients enrolled in the study, is presented. The graph also illustrates the reasons for exclusion from the study.

**Figure 4:**
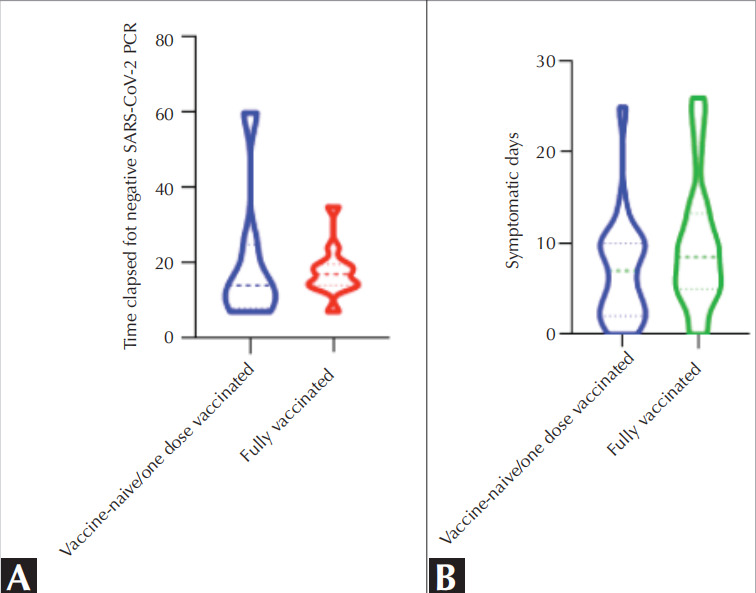
The course of SARS-CoV-2 infection in IEI patients. Time to negative SARS-CoV-2 PCR (A). Symptomatic days of COVID-19 infection in fully vaccinated and vaccine-naive/one-dose vaccinated patients (B).

The overall seroconversion rate among the study group after SARS-CoV-2 infection was 93% (n= 42). Seronegativity was observed in cases of BTK deficiency (n= 1) and immune dysregulation without a genetic etiology (n= 2). Interestingly, all seronegative patients were on IgRT. There was no significant difference in the levels of anti-SARS-CoV-2 antibodies among patients who received mRNA, inactivated vaccines, or combinations of vaccines ( Figure 2 B ). Due to the regular usage of IgRT, vaccine-naive patients showed similar titers of anti-SARS-CoV-2 antibodies compared to the fully vaccinated patients ( Table 2 ). Also, a slightly positive correlation between IgA and titers of anti-SARS-CoV-2 antibodies was detected (r= 0.3561, p= 0.021). It is worth mentioning that the hospitalization rate was higher among vaccine-naive or incompletely vaccinated patients (31.5%, 6/19) in comparison to fully vaccinated patients (23%, 6/26), underscoring the significance of vaccination within this vulnerable population ( Table 2 ). There was no mortality after immunization, while the dominant spreading variants were delta and omicron mainly caused the infection observed during the study period ( Figure 3 ). No statistically significant differences were observed between fully vaccinated individuals and those who were incompletely vaccinated or vaccine-naive, in terms of the duration required for SARS-CoV-2 PCR results to become negative and the number of days with COVID-19 symptoms ( Figure 4 A, B ).

Among fully vaccinated patients requiring hospitalization for SARS-CoV-2 infection, IgM and IgE levels were significantly lower than in non-hospitalized patients ( Table 3 ). The median duration of hospitalization in fully vaccinated patients was 4.5 (IQR= 2.75-6.5). The median duration of symptomatic days in hospitalized patients was 17.5 (IQR= 9.75- 27) and significantly higher than in non-hospitalized patients (p= 0.012), while the median time to negative PCR test results after infection was similar in hospitalized and non-hospitalized patients. In addition, the median titer of anti-SARS-CoV-2 antibodies in hospitalized patients was dramatically lower than in non-hospitalized patients (p= 0.016).

Finally, when we compared hospitalized and non-hospitalized patients regardless of vaccine status (fully vaccinated, incomplete regimens, and vaccine-naive), higher serum immunoglobulin levels and lower symptomatic days were observed in non-hospitalized patients ( Table 4 ).

**Table 1:** The demographic findings of COVID-19 positive IEI patients (n= 45)

Age (year, median, IQR)	35 (25.5-40)
Gender (male) (%)	24 (53.3)
Diagnosis*	n (%)
Predominantly antibody deficiency	28 (62.2)
Immune dysregulation disorders	13 (28.8)
Combined immunodeficiencies	1 (2.2)
Phagocyte defects	1 (2.2)
Complement deficiency	2 (4.4)
IgRT	n (%)
Intravenous route	
Subcutaneous route	
Prophylactic antibiotic	
Vaccination status	n (%)
Only mRNA vaccine (2, 3 doses)	
Only inactive vaccine (2, 3, 4 doses)	
Inactive + mRNA vaccine	
Vaccine-naive	
Probable COVID-19 variants	n (%)
Index virus	
Delta	
Omicron	
COVID-19 infection severity	n (%)
Asymptomatic-mild disease	
Moderate-severe disease	
Symptomatic days (median, IQR)	
Time to negative PCR test result (days, median, IQR)	
Hospitalization for COVID-19 infection	n (%)
Yes	12 (26.6)
No	33 (73.3)
Biochemical assessment	Median (IQR)
Trough IgG (mg/dL)	952 (746-1224)
IgA (mg/dL)	9 (9-59)
IgM (mg/dL)	38 (19-127)
IgE (mg/dL)	0.92 (0.2-7.8)
Anti-SARS-CoV-2 antibodies (U/mL, median, IQR)	975 (102.5-2500)
Lymphocyte subsets, absolute count (median, IQR)	
CD3^+^ T cells	1539 (114-2187)
CD4^+^ T cells	748 (462-965)
CD8^+^ T cells	768 (504-1027)
CD19^+^ B cells	160 (34-261)
CD16^+^ 56^+^ NK cells	84 (37-148)

IQR: Interquartile range, NK: Natural killer cells, IgRT: Immunoglobulin replacement treatment. *IUIS: International Union of Immunological Societies.

**Table 2:** Characteristics of fully-vaccinated versus vaccine-naive/one-dose vaccinated patients with COVID-19 infection

	Vaccine-naive/One-dose vaccinated	Fully-vaccinated	p
No of patients	19	26	
Gender (female/male)	7/12	14/12	0.25
Age (years, median, IQR)	31 (26-40)	35.5 (24.7-40.5)	0.712
Diagnosis			
PAD	12	16	
ID	5	8	
CID	0	1	0.69
Phagocyte defects	1	0	
Complement deficiency	1	1	
Anti-SARS-CoV-2 antibodies (U/mL, median, IQR)	402 (35.75-1983)	1307.5 (213-2500)	0.136
Biochemical assessment (median, IQR)			
IgG (trough) (mg/dL)	906 (736-1238)	1003 (772-1221)	0.48
IgM (mg/dL)	23 (19-83)	53.5 (19-91)	0.46
IgA (mg/dL)	11 (19-56)	9 (9-68.7)	0.91
IgE (mg/dL)	0.92 (0.2-7)	0.77 (0.2-13)	0.79
The severity of COVID-19 infection			
Asymptomatic-mild	15	19	0.651
Moderate-severe	4	7	
Hospitalization			
Yes/No	6/13	6/20	0.524
Duration of hospitalization (days, median, IQR)	1 (1-2)	1 (1-1.25)	0.605
Duration of negative PCR (days, median, IQR)	14 (8-25)	17 (14-19.7)	0.136
Duration of symptomatic days (median, IQR)	7 (2-10)	8.5 (4.7-13)	0.203
Interval between COVID-19 infection and antibody sampling (days, median, IQR)	357 (154.7-512.5)	70 (37.5-129.7)	
Interval between COVID-19 infection and last vaccination (days, median, IQR)	-	94 (36.7-161.25)	

PAD: Predominantly antibody deficiency, ID: Immune dysregulation disorders, CID: Combined immunodeficiencies, IQR: Interquartile range.

**Table 3:** Clinical and immunological characteristics of fully-vaccinated patients with hospitalization versus non-hospitalization

	Hospitalized (n= 6)	Non-hospitalized (n= 20)	p
Gender (female/male)	4/2	10/10	0.47
Age (year, median, IQR)	32.5 (23.5-35.25)	38 (25-42.75)	0.127
Diagnosis			
PAD	4	12	0.88
ID	2	6	
CID	0	1	
Phagocyte defects	0	0	
Complement deficiency	0	1	
Anti-SARS-CoV-2 antibodies (U/mL, median, IQR)	159 (81.7-948)	1568 (985.7-2500)	0.016
Biochemical assessment (median, IQR)			
IgG (trough) (mg/dL)	856.5 (626.7-991.7)	1069 (799-1351)	0.11
IgM (mg/dL)	19 (18.5-35.7)	71.5 (21.2-291.2)	0.015
IgA (mg/dL)	9 (9-513.7)	14 (9-104)	0.196
IgE (mg/dL)	0.2 (0.2-0.25)	2.63 (0.21-16.7)	0.009
Probably variants of SARS-CoV-2			
Index virus	1	0	
Delta	2	7	
Omicron	3	13	0.173
The severity of COVID-19 infection			
Asymptomatic-mild	1	18	
Moderate-severe	5	2	0.002
Duration of hospitalization (days, median, IQR)	4.5 (2.75-6.5)	-	
Duration of negative PCR after infection (days, median, IQR)	16 (14-55)	18 (14-19.5)	0.70
Duration of symptomatic day (median, IQR)	17.5 (9.75-27)	7 (4-11.5)	0.012
Interval between COVID-19 infection and antibody assay (days, median, IQR)	82.5 (53.2-246)	65.5 (23.7-111.2)	0.23
Interval between COVID-19 infection and last vaccination (days, median, IQR)	132 (15.7-194.2)	85 (41-157)	0.67
Vaccine brands			
Inactive vaccine (2, 4 doses)	3	8	
mRNA vaccine	1	6	-
Inactive + mRNA vaccine	2	6	

PAD: Predominantly antibody deficiency, ID: Immune dysregulation disorders, CID: Combined immunodeficiencies.

**Table 4:** Correlation between clinical parameters and ultrasound measurements

	Hospitalized (n= 12)	Non-hospitalized (n= 33)	p
Gender (female/male)	5/7	16/17	0.68
Age (years, median, IQR)	33 (27.7-38.2)	35 (24-41)	0.57
Diagnosis			
PAD	7	21	0.65
ID	5	8	
CID	0	1	
Phagocyte defects	0	1	
Complement deficiency	0	2	
Anti-SARS-CoV-2 antibodies (U/mL, median, IQR)	202 (36-1511)	1183 (112-2500)	0.25
Biochemical assessment (median, IQR)			
IgG (trough) (mg/dL)	800 (453-966)	1078 (823-1268)	0.002
IgM (mg/dL)	19 (19-36.5)	69 (19-173)	0.013
IgA (mg/dL)	9 (9-11.2)	19 (9-84.5)	0.019
IgE (mg/dL)	0.2 (0.2-0.35)	2.2 (0.2-10.3)	0.015
Probably variants of SARS-CoV-2			
Index virus	7	7	0.056
Delta	2	13	
Omicron	3	13	
Lymphocyte subsets (median, IQR)			
ALC/mm^3^	1600 (1325-2400)	2100 (1600-2800)	0.303
CD3^+^ T-cells	1299 (913-2248)	1625 (1215-2187)	0.96
CD4^+^ T-cells	491 (257-896)	820 (497-1129)	0.167
CD8^+^ T-cells	786 (457.2-975)	734 (504-1034)	0.78
CD19^+^ B cells	118 (26-220)	172 (34-299)	0.372
CD16^+^-56^+^ NK cells	699 (24-191)	100 (59-184)	0.291
Vaccination status			
Vaccine-naive/One-dose vaccinated	6	13	0.524
Fully-vaccinated	6	20	
Symptomatic days (median, IQR)	10 (9.25-22.5)	5 (2-10)	0.005
Duration of negative PCR (days, median, IQR)	16 (8-35)	14.5 (14-18.7)	0.50
Interval between COVID-19 infection and last vaccination (days, median, IQR)	132 (15.7-194)	75 (31-157)	0.51
Interval between COVID-19 infection and sampling antibody (days, median, IQR)	297 (73-538)	139 (62-262)	0.06

PAD: Predominantly antibody deficiency, ID: Immune dysregulation disorders, CID: Combined immunodeficiencies.

## DISCUSSION

In this report, we present the efficacy and immunogenicity of various brands of SARS-CoV-2 vaccines, as well as the outcomes of COVID-19 vaccine breakthrough cases in different groups of patients with IEI. Our study evaluated different types of IEI patients who encountered COVID-19 infection; 19 were vaccine-naive or incompletely vaccinated, and 26 were fully vaccinated. No deaths attributed to COVID-19 infection were recorded. Nevertheless, the rate of hospitalization was higher among individuals who were incompletely vaccinated or vaccine-naive, in comparison to the fully vaccinated group.

### The case fatality rate in the unvaccinated period

Research about the case fatality rate and the intensive care unit (ICU) admission rate due to COVID-19 infection in IEI patients showed a higher rate compared to similar ages of the general population. In a recently published study, which represented the largest review of its kind involving 649 patients with IEI, the rates of ICU admission and case fatality rate (CFR) following COVID-19 infection were identified as 16% and 9%, respectively ( 38 ). While in the general population, the CFR is 2.1% and increases with older ages [(range 0.5-18%), 0.3% <40 years to 13-20% in >80 years] (39,40). However, in Giorgia et al.’s study, the CFR was higher among IEI patients when evaluated for similar age ranges (7% for 20-40 years up to 36% for >70 years) ( 38 ). Our previous study also confirmed this result, which showed the CFR as 34% ( 8 ). In our current study, there was no mortality after vaccination, and the mortality rate before vaccination was higher in IEI patients when compared with the general population. The mortality was unrelated to the dominant spreading variant observed during the study period.

### Immune responses against COVID-19 Vaccines

The seropositivity rate following vaccination was between 20% and 83% ( 31 , 32 , 41 , 42 , 43 ). Adrian M. Shields ( 44 ) reported the seropositivity rate following two doses of SARS-CoV-2 vaccine with the Pfizer/ BioNTech BNT162b2 or Astra Zeneca as 54.8% in patients with primary and secondary immunodeficiencies, compared to 100% in healthy controls. Hagin et al. conducted a study involving 26 patients with primary heterogeneous immunodeficiencies who were administered the Pfizer/BioNTech BNT162b2 vaccine ( 32 ). Among them, 18 individuals out of the total 26 tested seropositive for the SARS-CoV-2 spike protein following the administration of two vaccine doses. Regarding vaccination strategies, our findings have demonstrated that mRNA vaccines elicited stronger antibody responses in comparison to inactivated vaccines. As anticipated, one out of the two patients with XLA exhibited seronegativity towards both vaccines and SARS-CoV-2 infection within our cohort. Additionally, two other individuals who tested antibody response following vaccination ( 44 ). According to our study, the median IgA, IgM, IgG, and IgE levels were significantly lower in hospitalized IEI patients than in non-hospitalized ones, regardless of the vaccination. Likewise, our study reported a significant positive correlation between IgA and titers of anti-SARS-CoV-2 antibodies. These results delineate that patients with high immunoglobulin levels can display better outcomes after COVID-19 infection.

### The severity of COVID-19 infection

The clinical spectrum of COVID-19 in COVID-19 patients varies from asymptomatic to mild symptoms to death, which could be related to the heterogenicity of IEI-studied groups ( 24 , 45 , 46 , 47 , 48 , 49 , 50 ). An Italian study conducted among patients with COVID-19 revealed that the case fatality rate (CFR) and cumulative incidence rate were comparable to those observed in the general population ( 51 ). However, in contrast to the general population, COVID-19-related deaths among COVID-19 patients have occurred at a lower median age ( 23 , 52 , 53 ). Similarly, in another cohort of COVID-19 patients, about 65% had a mild and asymptomatic COVID-19 infection severity ( 54 ). On the other hand, a cohort of IEI found that unvaccinated patients showed a higher hospitalization rate when compared with vaccinated subjects [40% unvaccinated vs. 4% vaccinated; odds ratio (OR) 15.0 (95% CI= 4.2-53.4); p< 0.001]. According to that study, the striking result was associated with a high hospitalization rate in patients with primary antibody deficiency during the unvaccinated period [odds ratio (OR) 14.7 (95% CI= 4.1-52.8); p< 0.001] ( 55 ).

In our study, severe COVID-19 disease was seen in 24.5% of the IEI patients, and the hospitalization rate for fully vaccinated patients was 23% and 31.5% for vaccine naive/incompletely vaccinated patients, respectively. After vaccination, there was a notable decrease in the hospitalization rate, which aligns with findings from other studies.

### Boosting Vaccination and Treatment Recommendations for Individuals with IEI and COVID-19 Infection

The immunogenicity of two or more vaccine doses in patients with heterogeneous IEI remains unclear, and we have limited data on booster SARS-CoV-2 vaccination. A study was conducted with 33 adults and children with IEI, 16 vaccinated with the Pfizer/ Biontech BNT162b2 and 17 with Coronavac receiving two or three vaccine doses. The seropositivity rates were 55% after the second dose and 74% after the third dose. As a result, they advised administering three vaccine doses to individuals with IEI in order to ensure optimal immunogenicity ( 56 ). A similar pattern was noted in another study, where sequential administration of up to three doses resulted in an elevation of protective antibody levels from 20.2 AU/mL to 145 AU/mL after the third dose ( 57 ). Their findings are in line with recent studies, demonstrating an increase of anti-SARS-CoV-2 antibodies in most patients with humoral immunodeficiencies ( 31 , 32 , 33 , 42 , 58 , 59 , 60 , 61 ). In our study, a substantial level of anti-SARS-CoV-2 antibodies reaching up to 2500 U/mL was identified, potentially attributable to the administration of two or more vaccine doses, further augmented by a SARS-CoV-2 infection.

The current COVID-19 vaccine guidelines for patients with PID include administering three initial doses of mRNA vaccine, followed by a booster dose, and subsequently a second booster dose four months after the last booster doses ( 62 ).

IgG products currently exhibit elevated anti-SARS-CoV-2 antibody titers when compared to earlier trials, owing to the broader vaccine coverage that has generated higher antibody levels than the infection itself in the general population, encompassing plasma donors as well ( 63 ). Furthermore, noteworthy seropositivity was observed in an XLA patient who was both COVID-19 naive and unvaccinated, likely attributed to the transmission of SARS-CoV-2 specific IgG from IgG products.

The National Institutes of Health treatment guidelines panel recommends the use of antiviral paxlovid and remdesivir for patients with PID who have moderate or severe COVID-19 infections. In Türkiye, the Minister of Health recommends remdesivir for patients with immunodeficiency. Nevertheless, in our study, only two patients received at-home therapy with remdesivir.

This study had certain limitations. Firstly, the absence of a healthy control group was a limitation; however, our findings aligned with existing research. Another constraint was the relatively small number of cases, although the inclusion of a diverse and rare disease group may justify the sample size. Additionally, T-cell response testing, considered a marker of viral neutralization, was not conducted. Nevertheless, the study provides insights into the efficacy and safety of various vaccine brands in patients with IEI. Furthermore, clear positive clinical outcomes were evident among these vulnerable patients following SARS-CoV-2 infection post-vaccination.

## CONCLUSION

In conclusion, we demonstrate adequate seropositivity in this heterogenous disease group and good outcomes of COVID-19 breakthrough in the vaccinated group with no death and 23% hospitalization rates. Our study provides sufficient data regarding the effectiveness and favorable clinical outcomes of various brands of COVID-19 vaccines in IEI patients.

## Acknowledgments

The authors thank Dem İlaç Limited Company for their unwavering support in providing the necessary kits, Elecsys^®^ Anti-SARS-CoV-2 S.

## Ethical Committee Approval

This study was approved by the Süreyyapaşa Chest Diseases and Thoracic Surgery Training and Research Hospital Scientific Work Committee (Decision no: 210, Date: 14.04.2021).

## CONFLICT of INTEREST

The authors declare that they have no conflict of interest.

## AUTHORSHIP CONTRIBUTIONS

Concept/Design: EK, BE, ÖA, Aİ, AÖ, SB

Analysis/Interpretation: EK, FMT, BE, AÖ, Aİ, FÖ

Data acqusition: EK, ÖA, FMT, EKA

Writing: EK, SB, FÖ

Clinical Revision: EK, ÖA, FMT, BE

Final Approval: EK, Aİ, BE, AÖ, FÖ, SB
